# Detoxification- and Immune-Related Transcriptomic Analysis of Gills from Bay Scallops (*Argopecten* *irradians*) in Response to Algal Toxin Okadaic Acid

**DOI:** 10.3390/toxins10080308

**Published:** 2018-07-28

**Authors:** Cheng Chi, Sib Sankar Giri, Jin Woo Jun, Sang Wha Kim, Hyoun Joong Kim, Jeong Woo Kang, Se Chang Park

**Affiliations:** 1 Laboratory of Aquatic Nutrition and Ecology, College of Animal Science and Technology, Nanjing Agricultural University, Weigang Road 1, Nanjing 210095, China; chicheng0421@126.com; 2 Laboratory of Aquatic Biomedicine, College of Veterinary Medicine and Research Institute for Veterinary Science, Seoul National University, Seoul 151742, Korea; giribiotek@gmail.com (S.S.G.); advancewoo@hanmail.net (J.W.J.); kasey.kim90@gmail.com (S.W.K.); hjoong1@nate.com (H.J.K.); kck90victory@naver.com (J.W.K.)

**Keywords:** harmful algal blooms, okadaic acid, *Argopecten irradians*, transcriptomic response, deep sequencing

## Abstract

To reveal the molecular mechanisms triggered by okadaic acid (OA)-exposure in the detoxification and immune system of bay scallops, we studied differentially-expressed genes (DEGs) and the transcriptomic profile in bay scallop gill tissue after 48 h exposure to 500 nM of OA using the Illumina HiSeq 4000 deep-sequencing platform. De novo assembly of paired-end reads yielded 55,876 unigenes, of which 3204 and 2620 genes were found to be significantly up- or down-regulated, respectively. Gene ontology classification and enrichment analysis of the DEGs detected in bay scallops exposed to OA revealed four ontologies with particularly high functional enrichment, which were ‘cellular process’ (cellular component), ‘metabolic process’ (biological process), ‘immune system process’ (biological process), and ‘catalytic process’ (molecular function). The DEGs revealed that cyclic AMP-responsive element-binding proteins, acid phosphatase, toll-like receptors, nuclear erythroid 2-related factor, and the NADPH2 quinone reductase-related gene were upregulated. In contrast, the expression of some genes related to glutathione S-transferase 1, C-type lectin, complement C1q tumor necrosis factor-related protein, Superoxide dismutase 2 and fibrinogen C domain-containing protein, decreased. The outcomes of this study will be a valuable resource for the study of gene expression induced by marine toxins, and will help understanding of the molecular mechanisms underlying the scallops’ response to OA exposure.

## 1. Introduction

Bivalves are among the most important commercially exploited marine species in China, sharing 75–80% of the total output of aquatic products in recent years [1]. Owing to their filter-feeding and sessile habits, worldwide distribution, and diversity of aquatic environments, bivalves are widely used as marine pollution bioindicators [2]. Scallop fisheries are mainly distributed along coastal areas of Japan, Korea, and North China [3]. In addition to their economic value, bivalves have always been studied as model species in toxicological investigation and as sentinel species in environmental monitoring programmes [4].

The frequent appearance of toxin-producing harmful algal blooms (HABs) in marine environments is a well-known worldwide problem [5]. HABs are well known for their potential to induce ecological damage, risk human health, and cause adverse effects to living marine resources [6,7]. Moreover, these HABs threaten aquaculture industries and may have deleterious effects on public health [8], because their phycotoxins may cause mass mortality of cultivated animals [9]. Shellfish toxins are the main marine phycotoxin, which includes amnaesic shellfish poisoning (ASP)-, paralytic shellfish poisoning (PSP)-, neurotoxic shellfish poisoning (NSP)-, diarrhetic shellfish poisoning (DSP)-, and azaspiracid shellfish poisoning (AZP) toxins [10]. These toxins may be taken up by humans eating shellfish contaminated with them, and lead to a series of neurological and gastrointestinal syndromes [6,7]. Okadaic acid (OA), representative of the DSP toxins, can be produced by species of the genera *Dinophysis* and *Prorocentrum* [11,12], and be accumulated in the shellfish adipose tissue [13]. This is the primary cause of acute DSP intoxication of human consumers, and harvesting bans causing huge economic losses to the shellfish aquaculture industry [14]. For example, Mouratidou et al. [15] reported maximum concentrations of 36 μg OA eq/g hepatopancreas in mussels from Thermaikos Gulf, Greece. OA is capable of binding to the active sites of protein phosphatases [16], inhibiting their activity and inducing tumorigenic and apoptotic processes [14,17]. Finally, it can lead to the hyperphosphorylation of many cellular proteins, metabolic deregulation, and genotoxic and cytotoxic damage [18]. When organisms are exposed to xenobiotics, short-term responses, such as changes in their immune response, and long-term effects on other biological parameters, including growth, ingestion and reproduction rates, and other metabolic processes may be observed [19]. Earlier investigations revealed that OA or *P. lima* exposure could induce haemocyte function damage and reduced survival in *Ruditapes decussatus* [20]. Huang et al. [11] reported that OA-producing *P. lima* caused oxidative stress, disorganization of cytoskeletons, and metabolic disturbance in mussels. In a previous work, we studied the toxic effects of OA exposure, up to 48 h, in bay scallops (*Argopecten irradians*). These included changes in glutathione (GSH), reactive oxygen species (ROS), malondialdehyde (MDA), and nitric oxide (NO) contents; lysozyme, acid phosphatase (ACP), lactate dehydrogenase (LDH), alkaline phosphatase (ALP), and superoxide dismutase (SOD) activity; total haemocyte counts (THC) and haemolymph total protein levels [8,12]. Overall, our previous work demonstrated that OA exposure increased oxidative stress, disrupted metabolism, modulated the immune response, and was toxic to physiological function in *A. irradians*. There are two resistance mechanisms that may counteract the effects of DSP in shellfish: detoxification pathways for the biotransformation or elimination of phycotoxins, and antioxidant metabolism to neutralize ROS induced by DSP exposure [21,22,23]. However, how scallops respond to OA toxicity, and the details of their detoxification process during acute OA exposure remain unclear, particularly the integral response at the transcriptional level. An understanding of the effects of OA exposure on the bay scallop is essential to establish effective measures to estimate its toxic potential. However, owing to the constraint of related genomic resources, a better understanding of the genetic and molecular mechanisms underlying the bay scallop response to sublethal concentrations of OA is yet to be elucidated.

De novo sequencing is an effective tool to obtain whole scallop transcriptome information. In this regard, the relatively low-cost/high-output Illumina HiSeq™ 4000 sequencing platform has found increasingly widespread use [24], having been applied to a growing number of aquatic organisms, including *Oryzias melastigma* [25], *Crassostrea gigas* [26], and *Chlamys farreri* [27], to study their responses to environmental stressors. Therefore, the aim of the present study was to obtain a better understanding of the molecular response of the bay scallop after exposure to OA. We specifically focused on the gill tissue of *A. irradians,* following exposure to 500 nM of OA for up to 48 h, since our previous studies found that this toxin induced oxidative stress, modulated the immune response, and was toxic to physiological function in *A. irradians* [8,12]. The gill was chosen as the target organ because it is the first organ in contact with OA during filtration [21]. Gills act as a defence barrier, because they play a crucial role in the filtration of suspended matter. Further, the gill was previously found to be directly affected by contact with toxic algae [21], and to have a high expression of putative immune-related genes [28]. Digital gene expression (DGE) analysis was performed with the Illumina HiSeq™ 4000 sequencing system, and then quantitative real-time PCR was conducted to verify differentially expressed genes (DEGs), which were selected according to the DGE analysis. The aim of the present work was to reveal the transcript abundance to facilitate a network of bay scallop genes enriched to regulate toxicological responses to OA exposure.

## 2. Results

### 2.1. Analysis of DGE Libraries

**Figure 1:**
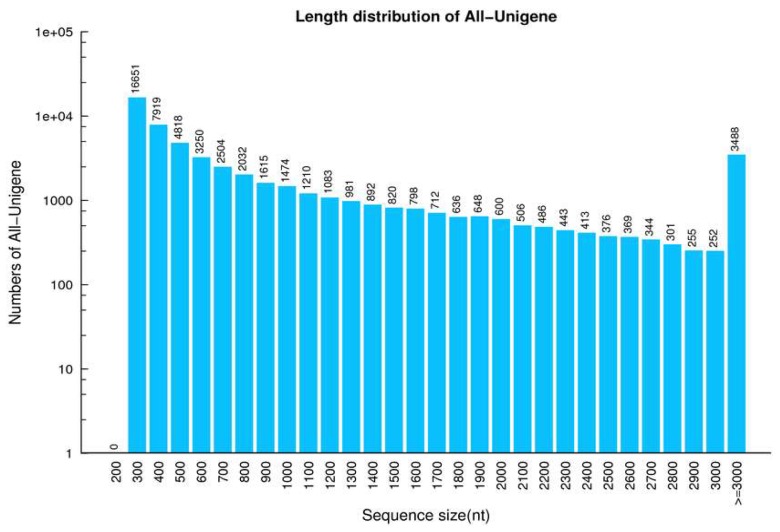
Distribution of all-unigenes in the bay scallop transcriptome.

Two DGE libraries comprising DNA from the gills of control and OA-exposed scallops were analysed using the Illumina Hiseq 4000 sequencing system. We removed adaptors from the reads, poly N, and low-quality reads from the raw data, and then generated 9.14 Gb of totally clean bases, comprising 45.92 and 45.92 Mb clean reads for control and OA-exposed cDNA libraries, respectively. The Q20 and GC percentages of the clean reads in the two cDNA libraries were 98.21% and 98.17% and 39.13% and 39.24%, for control and OA-exposed cDNA libraries, respectively (Table S1). Clean sequences from each library were assembled by the Trinity tool, thereby producing a total of 78,510 and 77,330 transcripts in the control and OA-exposed groups, respectively, which had mean sizes of 675 with N50s of 1234 for the control group and 733 bp with N50s of 1451 bp for the OA-exposed groups, respectively (Table S1). Finally, 55 876 unigenes were further merged by transcript sets from the two libraries (Table 1). The size distribution of the unigenes was as follows: 67.58% (37,759) were between 300 and 1000 bp; 20.54% (11,477) were between 1000 and 3000 bp; and 6.24% (3488) had lengths greater than 3000 bp in length, as shown in Figure 1.

### 2.2. Functional Annotation and Species Distribution

After assembly, functional annotation was carried out through seven functional databases for unigenes. A total of 49.31% of the total unigenes (27,555 unigenes) were annotated, of which 24,521 unigenes (43.88%) were aligned to the Nr database; 10,466 unigenes (18.73%) to Nt; 19,220 unigenes (34.40%) to Swiss-Prot, 18,523 unigenes (33.15%) to Kyoto Encyclopedia of Genes and Genomes (KEGG); 8800 (15.75%) unigenes to Clusters of Orthologous Group (COG); 18,533 (33.17%) unigenes to Interpro; and 4027 unigenes (7.21%) to Gene Ontology (GO), respectively.

**Figure 2:**
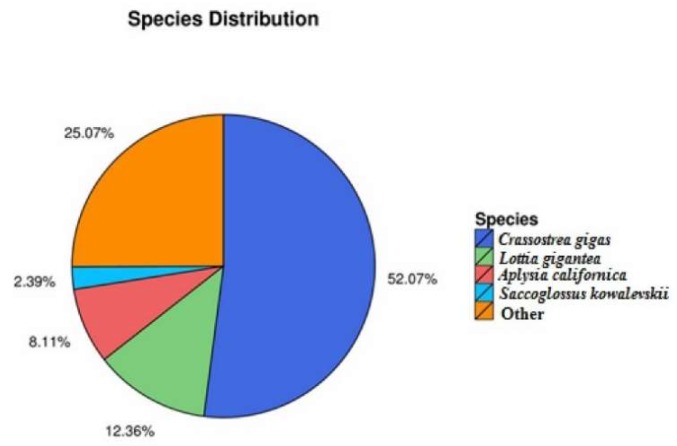
Annotated species and their distribution.

**Figure 3:**
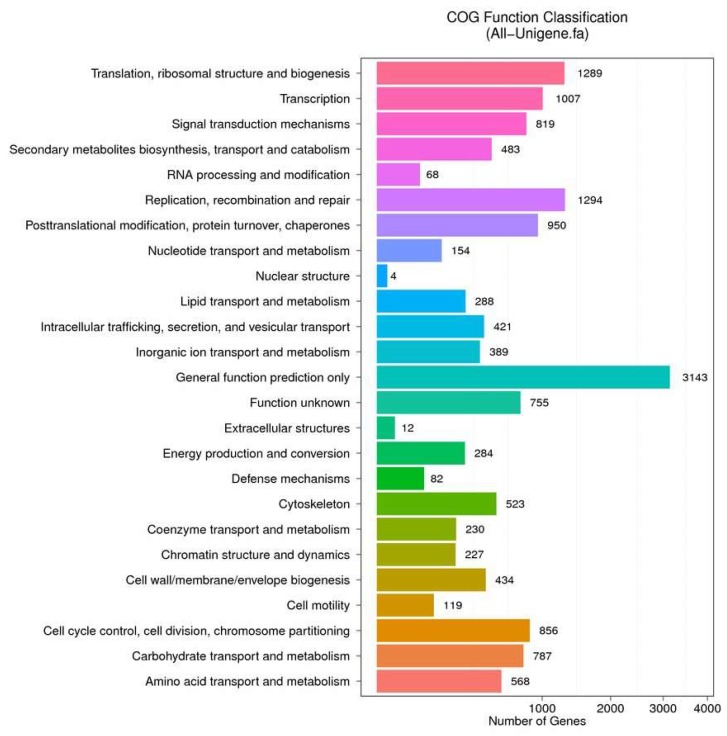
COG functional classification of All-unigenes.

The distribution of annotated species was statistically analysed with NR annotation, as shown in Figure 2. For functional classification, 15 186 unigenes were totally annotated to the COG database (Figure 3). The most frequently functional classifications were the following: 20.70% (3143) accounted for general function; 8.52% (1294) related to recombinant and repair; translation, 8.49% (1289); transcription, 6.63% (1007); post-translational-modification-related, 6.26% (950); cell-cycle-control-related, 5.64% (856), and signal-transduction-related, 5.39% (819).

### 2.3. Differential Gene Expression Analysis

**Figure 4:**
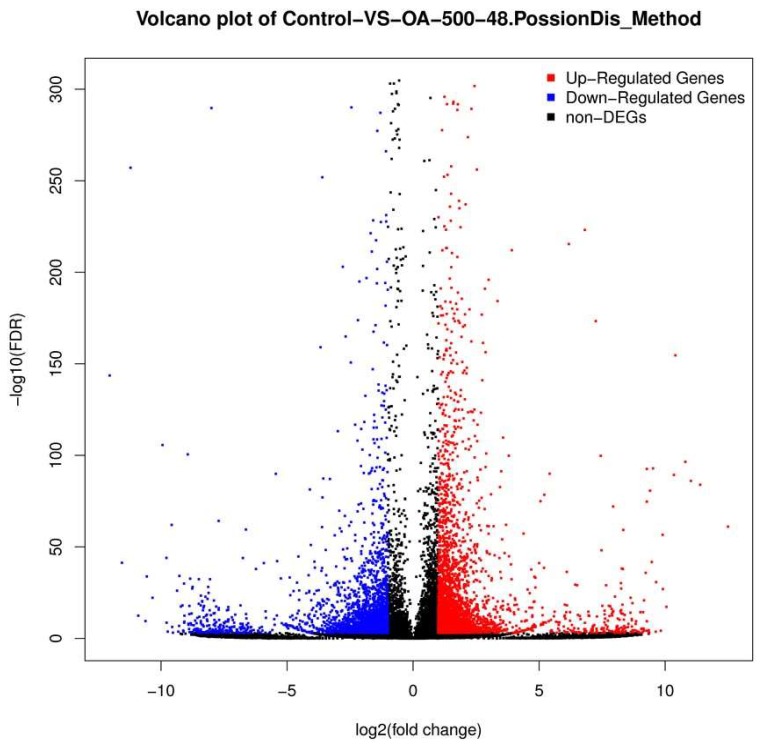
Gene transcription profile of the control (CN) and the OA-exposed group (OA) libraries. Blue points represent downregulated genes. Red points represent upregulated genes. Black points represent non-differential expression genes.

**Figure 5:**
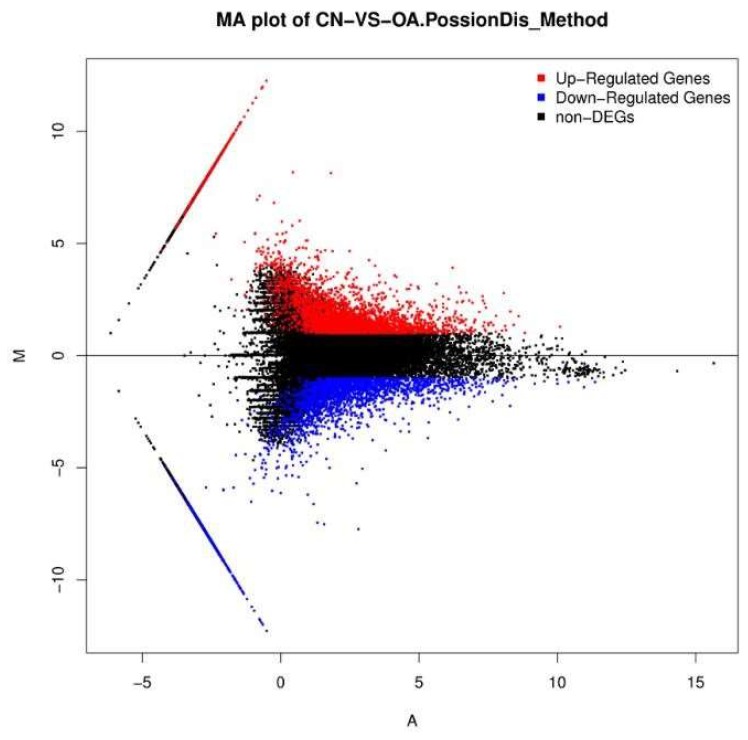
M (log ratio) and A (mean average) (MA) plot of DEGs of the control (CN) and the OA-exposed group (OA) libraries. *X*-axis represent value A (log2 mean expression level). *Y*-axis represents value M (log2 transformed fold change). Red points represent upregulated DEG. Blue points represent downregulated DEG. Black points represent non-DEGs.

The unigene expression levels were calculated using the Fragments Per Kilobase Million (FPKM) method (Figure 4 and Figure 5) to identify the genes’ differential expression between the control and OA-treated groups. A total of 5825 unigenes with different expression levels (with over two-fold changes, and false discovery rate (FDR) ≤ 0.001) between the control and OA-exposed groups were identified. Of these, 3204 were upregulated genes, while 2621 were downregulated genes (Table S2).

### 2.4. Enrichment and Pathway Analysis

**Figure 6:**
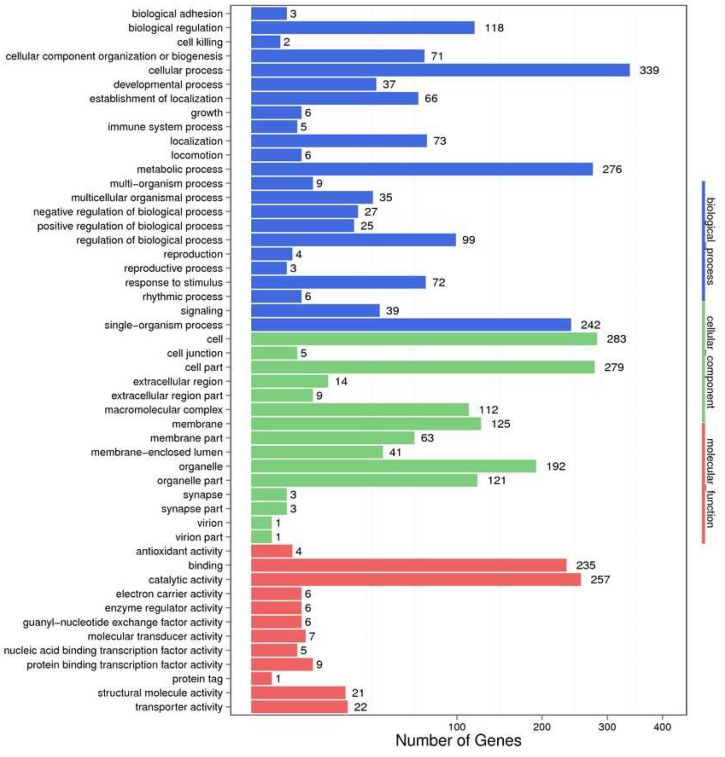
GO classification of differentially expressed gene (DEGs). *X*-axis represent the GO term.

In order to identify their function, all the DEGs were mapped to the GO database. A total of 44 functional groups in the DEGs were substantially enriched compared with the genomic background (Figure 6). Genes in the OA-exposed scallop related to the terms ‘metabolic process’, ‘cellular process’, and ‘catalytic activity’ were dominant. Biological process and cellular components were found to be the most-represented known genes, followed by molecular function.

**Figure 7:**
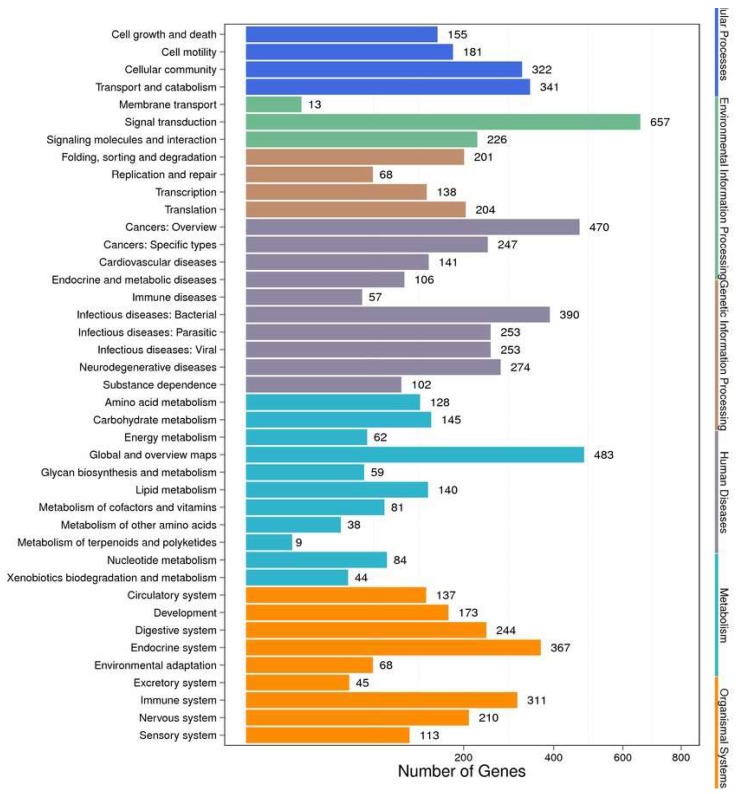
Pathway classification of DEGs. The *X*-axis shows the number of DEGs. The *Y*-axis shows the pathway name.

**Figure 8:**
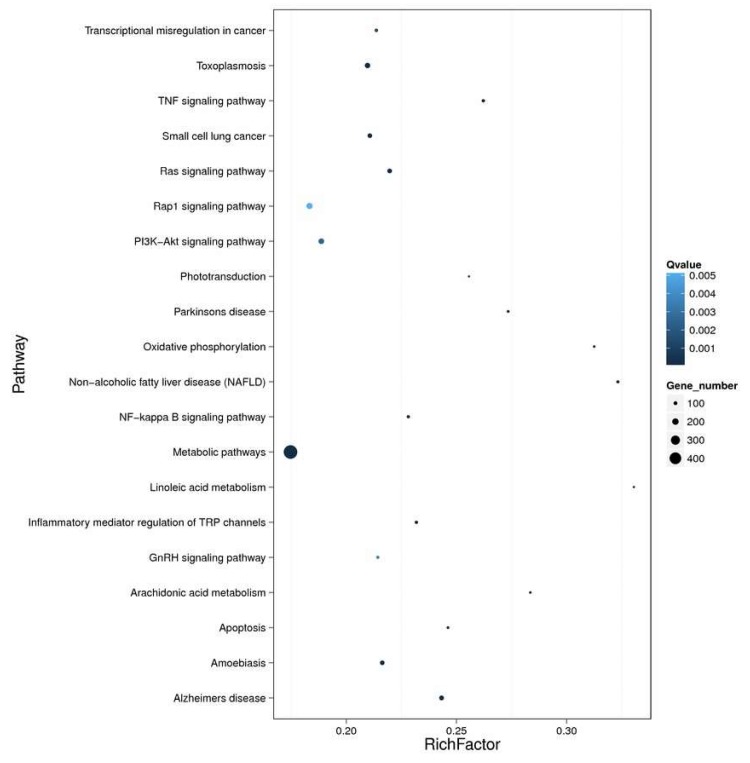
Enrichment of DEGs and pathways. The *X*-axis indicates enrichment factor and the *Y*-axis indicates the pathway name. Coloring indicates the *q* value (high: white, low: blue), the lower *q* value indicates the more significant enrichment. The point size indicates the DEG number (more: big, less: small).

Markedly-enriched signal transduction and metabolic pathways were identified using KEGG enrichment analysis of the DEGs. A total of 3389 DEGs were aligned at 299 pathways in the KEGG database, and 74 metabolic pathways were significantly (corrected *p* value < 0.05) over-represented. The pathway classification results are shown in Figure 7, and the pathway functional enrichment results in Figure 8. Among these, the expression patterns of DEGs throughout OA exposure, which involved detoxification, and immunology in mechanisms against biotoxins were further analyzed on the bases of GO and KEEG analyses. The expression of genes related to the immunology and detoxification responses such as cyclic AMP-responsive element-binding proteins, acid phosphatase, toll-like receptors, nuclear factor erythroid 2-related factor, NADPH2: quinone reductase, cytochrome P450 3A64 and 3A80 increased under exposure to OA (Table 2). In contrast, the expression of some genes related to glutathione S-transferase 1, C-type lectin, complement C1q tumor necrosis factor-related protein, Superoxide dismutase 2 and fibrinogen C domain-containing protein decreased.

### 2.5. Identification of Genes Related to OA-Induced Stress Response

**Figure 9:**
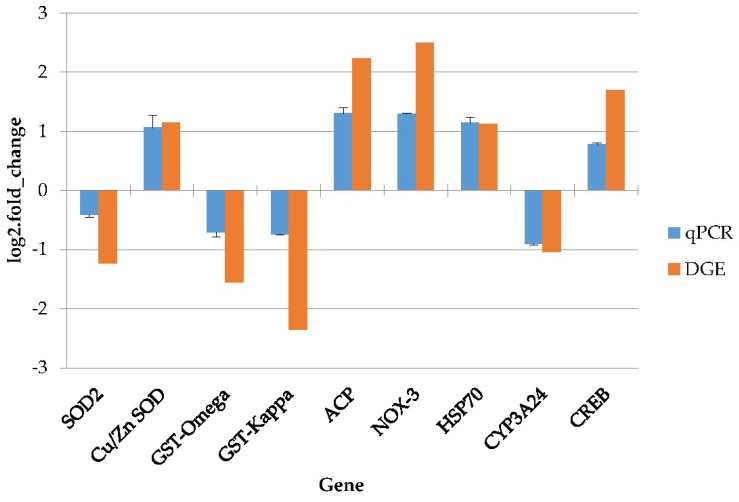
Results of the qPCR analysis. The *y*-axis represents the gene expressed log2 (fold change) and the *x*-axis is the gene name. *SOD2* = superoxide dismutase 2, *Cu/Zn SOD* = copper and zinc superoxide dismutase, GST = glutathione-S-transferase, ACP = acid phosphatase, *NOX*-3 = NADPH oxidase 3, *HSP70* = heat shock protein 70, *CYP3A24* = cytochrome P450 3A24, *CREB* = cyclic adenosine monophosphate responsive element binding protein.

The real-time quantitative PCR (qPCR) technique was used to detect the relative expression levels of nine genes, which are immunology-, detoxification- and antioxidant-ability-related genes with high expression, from the DGE libraries. Four of these genes were suppressed and the others were induced. The melting-curve analysis of each gene performed by qPCR suggested a single product. The qPCR results were compared with those from the DGE analysis. As shown in Figure 9, nine genes followed a concurrent trend between qPCR analysis and DGE library, and the correlation coefficient was calculated as 0.95 (*p* value < 0.001).

## 3. Discussion

Okadaic acid (OA), as a representative of DSP toxins, can accumulate in bivalves and induce diarrheic shellfish poisoning in mammals [29]. OA has been reported to be cytotoxic in several cell lines (human monocytic U-937 cells; two epithelial tumour lines, HeLa and KB; neuroblastoma cell line Neuro-2a; neuroblastoma × glioma hybrid cell line NG108-15; breast cancer cell line MCF-7) as an efficient inhibitor of serine/threonine phosphatases [30,31,32]. Earlier, we reported that OA exposure could affect a variety of innate immune responses (e.g., THC, total protein level, ALP, ACP, and lysozyme activities,) and physiological responses (e.g., SOD and LDH activity, ROS, NO and MDA and GSH content) in the haemolymph of scallops, and can even induce oxidative stress and disrupt metabolism in bay scallops [8,12], rendering them sensitive to OA exposure. Previous studies have demonstrated the adverse impacts of the toxin OA on other marine bivalves [11,20]; however, the molecular response of these bivalves to OA is not well characterized. In the light of our earlier studies, the results of this transcriptome information could improve the description of the acute toxicity of high concentrations of OA for some physiological and biochemical processes and provide directions and insights for future studies involving biotoxicity models in scallops. Moreover, in the present study, the calculation and normalization methods of both analyses are different, although they report transcript abundances as fold-changes relative to the control [1]. The RNA-seq expression values employ Reads Per Kilobase Million (RPKM) for calculation [33], while qPCR fold-change values employ the mean normalized expressions method and incorporated reference gene to calculate [34]. In the present investigation, both methods were used for transcript quantification. The same directions of change and a similar magnitude of the fold-change in abundance confirmed the accuracy and reliability of the DGE data. To our knowledge, the present investigation is the first to reveal the transcriptomic responses of scallops after OA exposure using deep-sequencing technology.

Highly conserved heat shock proteins (HSPs), including *HSP60*, *HSP70*, and *HSP90*, could be synthesized or secreted rapidly by cells as soon as they experience stressed [3]. Therefore, HSPs have been widely considered as effective biomarkers of exogenous stimuli or as biomonitoring tools to identify the effects of environmental pollution in aquatic animals, including bivalves [3]. Our present investigation showed that the relative expression of *HSP70*, which was validated by qPCR, was strongly increased in the gills of bay scallop up to 48 h exposure to OA. Similarly, a previous study revealed that upregulated *HSP70* expressed transcripts were identified in the mussel *Mytilus galloprovincialis* after exposure to OA stress [14]. In other investigations, the detection of *HSP70* by immunoblotting and expression analysis of *HSP70* mRNA was used to indicate marine contamination observed following exposure to heavy metals in *Dreissena polymorpha* [35], to hydrocarbon in *Crassostrea gigas* [36], to sub-lethal concentrations of quaternium-15 in *M. galloprovincialis* [37], and to cadmium in the gills of *Ostrea edulis* [38]. Therefore, in the present investigation, the upregulation of *HSP70* mRNA in the gills of bay scallops also appears to be a helpful marker for toxic effects.

The genes encoding detoxification enzymes play crucial roles in bivalves after being stimulated by a variety of exogenous stimuli, including drugs, toxicants, and chemical carcinogens [3]. Among the DEGs detected in the present study, certain detoxification-related genes were identified. The cytochrome P450 (*CYP450*) family is an essential family of enzymes related to the primary or phase I metabolism of xenobiotics, including pesticides and toxins [3,39]. Many exogenous stimuli may impact the metabolism, and then activate or suppress the activity of *CYP450* to clean exogenous stimuli [11]. A subset of cytochrome P450 enzymes, which are linked to detoxification and resistance, were involved in transforming liposoluble toxic chemicals into hydrosoluble substances that are easily eliminated [11,40]. Our results clearly showed that OA provoked the differential expression of *CYP1A5* and *CYP3A24*, which were downregulated, whereas *CYP3A4* and *CYP3A80* were upregulated. This is consistent with a previous study that reported OA-exposure-induced expression of *CYP450* mussel gills, which suggests that *CYP450* participates in the process of OA elimination [3]. Guo et al. also [41] reported that human recombinant cytochrome *CYP3A4* could eliminate OA by generating oxidized products. Accordingly, *CYP3A4* and *CYP3A80* may participate in the process of accelerating the biotransformation of OA and facilitating its excretion in bay scallops when exposed to OA. ATP-binding cassette (ABC) transporters are a family of transmembrane proteins that can transport a variety of strGSTucturally diverse substrates across biological membranes in an ATP-dependent manner [11]. In mammalian tumor cells, they are responsible for a multidrug resistance phenotype. Moreover, in aquatic organisms, they are responsible for a multixenobiotic resistance phenotype by exporting xenobiotics out of the cells or by facilitating the sequestration of toxins within specialized cells or organelles, effectively segregating them away from vulnerable protein and DNA targets [11]. In our present study, we found that *ABCB10*, *ABCC5*, and *ABCC1* were upregulated in bay scallops after 500 nM OA exposure. These results are consistent with a previous study showing that ABC transporters in mussels were upregulated after exposure to *P. lima*. Huang et al [42] also found that the expression level of a P-glycoprotein gene (*P-gp*), belonging to the family of ATP-binding cassette (ABC) transporters in the gills of *Perna viridis,* increased significantly after exposure to *P. lima*. These phenomena suggest the possible role of ABC transporters in OA detoxification.

Nicotinamide adenine dinucleotide phosphate-oxidases (NADPH-oxidases) are enzymes completely devoted to ROS production [43]. The family of NADPH-oxidases comprises trans-membrane proteins that transfer electrons across biological membranes. Owing to their involvement in ROS production, NADPH-oxidases play crucial roles in various physiological mechanisms which include host defence, gene expression, cellular signalling, apoptosis, and oxidative stress [44]. The NADPH oxidase is composed of six homologues of the cytochrome subunit (NOX1, NOX3, NOX4, NOX5, DUOX1, and DUOX2), and increased NOX activity also induces a series of pathologies [44]. Cai et al. [1] found that benzo[a]pyrene (BaP) exposure caused the upregulation of NADPH transcript in *Chlamys farreri* after three days. The findings of the present study indicated a greater abundance of *NOX-3* transcripts in the gills of scallops exposed to OA, suggesting that it induces the activation of the NADPH oxidases, thereby generating more ROS and even cell damage.

The detoxification and biotransformation of exogenous compounds also rests on Phase II and Phase III reactions [1]. Glutathione-*S*-transferase (GST), which is a kind of Phase II enzyme, could catalyse the endogenous and exogenous compounds combining with glutathione (GSH) [1,45]. Our previous field studies have shown that GSH levels in the haemolymph of *A. irradians* exposed to 500 nM OA decreased sharply at 48 hpe [12]. Consistently, in the present study, the expression of *GST* mRNAs, including *GST1*, *GST2*, *GST-A*, *GST-Theta-1*, *GST-Omeaga*, and *GST-Kappa*, in the DGE library decreased in the gills of *A. irradians* exposed to OA compared to the control group. This is consistent with previous studies showing that the expression of *GST-pi* was significantly down-regulated in the digestive gland of *M. galloprovincialis* in response to toxic dinoflagellate *Prorocentrum lima* (1000 cells/L) for 48 h [46]. These results suggest that the expression level of GST was attenuated by 500 nM OA exposure, which weakened the detoxification or antioxidant capacity of the OA-exposed scallops. SOD is a crucial gene belonging to the antioxidant defence system. It can eliminate the ROS, which can induce lipid peroxidation processes and ultimately lead to DNA damage [47]. We previously reported that *Mn SOD* expression levels in the haemolymph of OA-exposed bay scallops decreased significantly after 48 hours post-exposure [8]. These observations are in agreement with the results of the present study, verified by qPCR, showing that the *SOD2* expression levels in gills were downregulated after 48 h exposure to OA. However, we found that the expression of the *Cu/Zn SOD* mRNA was clearly induced, indicating that OA exposure could induce the expression level of *Cu/Zn SOD* in the gills when the scallops are exposed to up to 48 h 500 nM levels of OA. Additionally, it might be plausible that the downregulation of GST is partially compensated by the upregulation of *Cu/Zn SOD*, since both enzymes use the same substrate [46,48,49].

Cyclic adenosine monophosphate responsive element binding-protein (CREB) plays a pivotal role in the immune response. OA stimulation was found to enhance the levels of phosphorylated-CREB [50]. The expression of these genes is essentially regulated by the phosphorylation state of CREB, since phosphorylation is necessary for CREB to bind to the cAMP response element in the promoter of several early response genes [50]. This result is in accordance with a previous study showing that OA was able to induce CREB expression in mussels [50]. Acid phosphatase (ACP) is a kind of essential hydrolytic enzyme in phagocytic lysosomes [51]. In the present research, we found that the *ACP* mRNA expression increased in the gills of OA-exposed bay scallops. Nevertheless, in an earlier investigation, we demonstrated that OA exposure suppressed the ACP levels in the haemolymph of bay scallops, indicating that although OA could induce ACP expression, it might also affect the assembly, folding, or modification of the ACP, leading to a deficiency in the elimination of pathogens or phagocytized microorganisms in the OA-exposed gills.

In conclusion, we present here broader research into the OA-responsive genes, such as the Toll-like receptor, ATP-binding cassette, cyclic AMP-responsive element-binding protein, cytochrome P450 and Cu-Zn superoxide dismutase related genes, that show differential expression in the bay scallop, suggesting participation in the resistance to OA toxicity. These genes are related to a series of detoxification and immune processes in the response to OA. The present investigation not only reveals the transcriptional complexity of the response to OA stimulation in scallops, but also suggests the possibility of identifying the genes implicated in regulating the bivalves’ tolerance or the elimination of algal toxin stress. However, it remains unclear whether these immune responses are directly stimulated by abiotic factors or whether OA exposure just facilitates the opportunistic attack of pathogens present in the scallops’ microbiota [14]. Illumina next-generation sequencing technology provides a good resource to explain the immune- and detoxification-associated molecular mechanisms triggered in the bay scallop to endure the toxic effects of OA. Furthermore, it supplements and reinforces the results from our previous investigations, from which a strong cause and effect relationship between OA and the differential expression of immune- and detoxification-associated factors in the bay scallop were established. These results will be useful to develop potential countermeasures to manage the toxic effects of OA on exploited bivalve resources.

## 4. Materials and Methods

### 4.1. Maintenance of Scallops

Bay scallops *A. irradians* (weight: 46.02 ± 2.67 g; shell length: 60–70 mm) were procured in a wholesale market in Seoul, South Korea. To acclimate them to laboratory conditions, these scallops were kept for 2 weeks in 800-L tanks containing filtered and aerated seawater, with a temperature of 10 ± 1 °C and a salinity of 30 ± 0.1 psu [8]. They were fed with a commercial shellfish diet (Instant Algae® Shellfish Diet, Campbell, CA, USA) at a rate of approximately 1.2 × 10^10^ algae cells/scallop/day [8]. Half the seawater volume was daily renewed.

### 4.2. Okadaic Acid Exposure and RNA Extraction

In total, 120 scallops were divided in two groups, i.e., control and OA-exposure groups. Each group consisted of 60 scallops distributed in 3 replicate tanks with 20 scallops each. Okadaic acid (OA) (92–100% HPLC purified) was purchased from Sigma-Aldrich, USA and stored at 4 °C until use. To prepare the stock solution, OA was dissolved in 1 mL of dimethyl sulfoxide (DMSO; Sigma-Aldrich, St. Louis, MO, USA). The final concentration of OA in the OA-treated group was kept at 500 nM [8]. The scallops in the control group were treated with an equal volume of DMSO, with a final concentration of 0.0125‱DMSO in each tank [8]. After 48 h of OA exposure, six scallops were collected from each tank (i.e., 6 scallops × 3 replicates = 18 scallops per group) and maintained on ice. Scallop gills were dissected, stored in 1 mL TRIzol reagent (Invitrogen, Waltham, MA, USA), and frozen at −80 °C until use. Samples from 6 scallops were pooled for each replicate for RNA extraction [14]. Total RNA was extracted using TRIzol (Invitrogen, Waltham, MA, USA) following the manufacturer’s instruction. Agarose gels (1%) electrophoresis was preformed to monitor the RNA contamination and degradation [52]. The RNA purity and contamination was checked with a NanoPhotometer^®^ spectrophotometer (IMPLEN, Westlake Village, CA, USA) and a Qubit^®^ RNA Assay Kit and a Qubit^®^ 2.0 Flurometer (Life Technologies, Carlsbad, CA, USA) respectively [52]. RNA integrity was measured using the RNA Nano 6000 Assay Kit of the Agilent Bioanalyzer 2100 system (Agilent Technologies, Santa Clara, CA, USA) [52].

### 4.3. Library Preparation and Illumina Sequencing

After treating the total RNA extract sample with DNase I, 200 ng were purified with oligo-dT beads. In brief, total RNA and RNA Purification Beads (Illumina, San Diego, CA, USA) were incubated and resuspended in Elution Buffer (Illumina, San Diego, CA, USA). The mRNA was eluted from the beads, and then incubated to rebind the beads after adding Bead Binding Buffer (Illumina, San Diego, CA, USA). Finally, Fragment Buffer was used to fragment poly (A)-containing mRNA into small pieces. The mRNA fragments were used as templates during the cDNA synthesis. First-strand cDNA was synthesized by reverse transcription using First Strand Master Mix (Illumina, San Diego, CA, USA) and Super Script II (Invitrogen, Waltham, MA, USA). The conditions for the reverse transcription reaction were: 25 °C for 10 min; 42 °C for 50 min and 70 °C for 15 min. Next, the second-strand cDNA was synthesized at 16 °C for 1 h using Second Strand Master Mix (Illumina, San Diego, CA, USA). Then, the ds cDNA was separated from the second strand using AMPure XP beads (Agencourt, Beverly, MA, USA). The remaining overhangs were converted into blunt ends using an End Repair Mix. Next, after adding the A-Tailing Mix, the mixture was incubated at 37 °C for 30 min. The Adenylate 3′Ends DNA, RNA Index Adapter and Ligation Mix were combined and the ligate reaction incubated at 30 °C for 10 min to perform the A ligation reaction. AMPure XP Beads were used to purify the end-repaired DNA. In order to enrich the cDNA fragments, several rounds of PCR amplification were performed by adding PCR Primer Cocktail and PCR Master Mix. The AMPure XP Beads were used to purify the library fragments to select cDNA fragments of 260 bp in length. The final library quantified (qPCR) by loading 1 µL of resuspended construct on an Agilent Technologies 2100 Bioanalyzer using a DNA-specific chip (Agilent DNA 1000). For cluster generation, the qualified and quantified libraries were first amplified within the flow cell on the cBot instrument (HiSeq^®^ 4000 PE Cluster Kit, Illumina, San Diego, CA, USA).

For paired-end sequencing, the clustered flow cell was then loaded onto the HiSeq 4000 Sequencer (HiSeq^®^ 4000 SBS Kit, Illumina, San Diego, CA, USA) with 100 bp which was the recommended read length. The library preparation and Illumina sequencing were performed by the Beijing Genomics Institute (BGI) (Hong Kong, China).

### 4.4. De Novo Transcriptome Assembly

In order to remove adaptors from the reads, low-quality reads, and reads in which unknown bases (N) comprised more than 5% of the read, raw Illumina paired-end reads were filtered using the SOAPnuke software (version: v.1.5.6, Beijing Genomics Institute, Shenzhen, China, https://github.com/BGI-flexlab/SOAPnuke,). Post-filtered reads were stored in the FASTQ format [53]. To obtain unigenes, clean reads were assembled using the Trinity software (version: v2.0.6, Trinity Software, Arlington, Tx, USA) [54]. The resulting sequences assembled using Trinity were referred to as transcripts. Gene family clustering was then carried out using TGICL (TIGR Gene Indices clustering tools) to obtain the final unigenes, which were classified to two categories: clusters and singletons. The former were labeled by the prefix ‘CL’, followed by the cluster ID. The latter were indicated by the prefix ‘unigene’.

### 4.5. Gene Annotation and Analysis

Identification and functional annotation of all unigene sequences were carried out in seven functional databases (*e*-value < 10^−5^): Nr, Nt, GO, COG, KEGG, Swiss-Prot, and Interpro databases. Blast (version: v2.2.23, NCBI, Bethesda, MD, USA, http://blast.ncbi.nlm.nih.gov/Blast.cgi) [55] was used to align the unigenes to NT, NR, COG, KEGG, and SwissProt to obtain annotations. Blast2GO (version: v2.5.0, BioBam, Valencia, Spain, https://www.blast2go.com) [56] used NR annotations to obtain GO annotations, and InterProScan5 (version: v5.11-51.0, EMBL-EBI, Hinxton, UK, https://code.google.com/p/interproscan/wiki/Introduction) to obtain InterPro annotations.

### 4.6. Differential Expression Analysis

Bowtie v2.2.5 was devoted to map the high-quality reads to the reference unigene sequences [57], and then calculate the gene expression levels, which were determined using RSEM (version: v1.2.12, http://deweylab.biostat.wisc.edu/RSEM) [58]. DEGs were detected based on a Poisson distribution using PossionDis, as described by Audic and Claverie [59]. The unigene expression level was calculated following the fragments per kilobase million (FPKM) formula. A false discovery rate (FDR) of 0.001 and a two-fold change were selected as the thresholds for significantly differential expression.

### 4.7. GO and KEGG Enrichment Analysis of Differentially Expressed Genes

DEGs were classified according to the official classification on the basis of the GO annotation results. Pathway functional enrichment was also carried out by the *R*-function *phyper.* The *p* value calculating formula in the hypergeometric test was as Equation (1):

(1) $$P=1-\sum_{i=0}^{m-1}\frac{\left(\begin{array}{c} M\\ i \end{array}\right)\left(\begin{array}{c} N-M\\ n-i \end{array}\right)}{\left(\begin{array}{c} N\\ n \end{array}\right)}$$

FDR was calculated for each *p* value, and in general, the terms for which FDR did not exceed 0.001 were defined as significantly enriched.

### 4.8. Quantitative Real-Time PCR Validation

The expression of nine genes, which were singled out for the validation of the DGE data, was performed by qPCR. *β-actin* was used as a house-keeping gene [8]. cDNA synthesis was performed with 500 ng of DNase-treated RNA by using a PrimeScriptTM RT Reagent Kit (TaKaRa Bio, Kyoto, Japan). All qPCR reactions were carried out using SYBR Premix Ex TaqTM Perfect Real-Time Kits (TaKaRa Bio, Japan) with a QiagenRotor-Gene Q RT-PCR Detection System (Qiagen, Hilden, Germany). PCR primers, listed in Table S3, were designed using the Primer 5 software (version: v.5, PREMIER Biosoft, Palo Alto, CA, USA) based on transcriptome sequences. The reaction mixture consisted of 1 µL cDNA (50 ng), 1 µL of the forward and reverse primers (10 µM), and 6.25 µL of SYBR Premix Ex TaqTM. To ensure that the final volume of the reaction mixture was 12 µL, ultra-pure water was added. The following reaction conditions were maintained for extension: 94 °C for 2 min, followed by 40 cycles of 94 °C for 20 s, 58 °C for 30 s, and 72 °C for 40 s [8]. In order to eliminate the possibility of primer dimer formation or non-specific amplifications, a melting curve analysis was carried out after the amplification phase [8]. A standard curve was constructed from serial dilutions of the cDNA sample and drawn by plotting the natural log of the threshold cycle (Ct) against the number of molecules [8]. Standard curves for each gene were prepared in duplicate and triplicate to obtain a reliable measure of the amplification efficiency [8]. The amplification efficiencies were between 90% and 110%, and the correlation coefficients (*R*2) of all standard curves were >0.99. The relative expression ratios of the target genes were calculated using the method described by M.W. Pfaffl [60]. In all cases, PCR was carried out in triplicate. Statistical analysis was carried out using the statistical software SPSS 19.0 (version: 19.0, IBM Corp., Armonk, NY, USA, 2017). The differences were determined using the LSD test, with *p*-values < 0.05 indicating statistical significance. Values were expressed as the arithmetic mean ± standard deviation (SD).

## Figures and Tables

**Table 1:** Quality metrics of unigenes.

Sample	Total Number	Total Length	Mean Length	N50	N70	N90	GC(%)
Control	51,465	41,105,722	798	1411	704	302	39.48
OA-treated	49,453	43,129,157	872	1646	803	318	39.63
All-unigene	55,876	53,465,429	956	1840	960	345	39.42

N50: a weighted median statistic within which 50% of the Total Length is contained in unigenes greater than or equal to this value. GC (%): the percentage of G and C bases in all unigenes.

**Table 2:** Detoxification and immune-related differentially expressed genes (DEGs) in bay scallop gills regulated after up to 48 h exposure to 500 nM OA.

Function	Transcript	Log2 (Fold Change) (RNAseq)	Regulation
Immune system	C-type lectin superfamily 17 member A	−4.255	Down
	C-type lectin domain family 4 member E	−3.507	Down
	Complement C1q tumor necrosis factor-related protein 2	−4.791	Down
	Fibrinogen C domain-containing protein 1	−2.100	Down
	Toll-like receptor 4	2.880	Up
	Toll-like receptor 13	1.347	Up
	Acid phosphatase	2.238	Up
	NADPH oxidase 3	2.493	Up
Detoxification	ATP-binding cassette, subfamily C, member 1	1.773	Up
	ATP-binding cassette sub-family B member 10	1.165	Up
	ATP-binding cassette, sub-family C member 5	1.280	Up
	Cyclic AMP-responsive element-binding protein	1.953	Up
	Nuclear factor erythroid 2-related factor 2	1.231	Up
	NADPH2:quinone reductase	1.677	Up
	Cytochrome P450 3A80	1.207	Up
	Cytochrome P450 3A64	1.783	Up
	Cytochrome P450 1A5	−1.686	Down
	Cytochrome P450 3A24	−2.315	Down
	Superoxide dismutase Cu-Zn family	1.139	Up
	Superoxide dismutase 2	−1.126	Down
	Glutathione S-transferase 1	−1.552	Down
	Glutathione S-transferase 2	−2.511	Down
	Glutathione S-transferase omega	−1.775	Down
	Glutathione S-transferase theta-1	−1.254	Down
	Glutathione S-transferase A	−1.218	Down
	Glutathione S-transferase kappa	−2.356	Down

## Supplementary Materials

The following are available online at http://www.mdpi.com/2072-6651/10/8/308/s1, Table S1: Summary of sequencing reads after filtering, and quality metrics of transcripts, Table S2: The differential expression unigenes (with higher than two-fold changes, and FDR≤ 0.001) between the control and the OA-treated groups. Table S3: All primers used in the validation analysis, The accession number for our raw dataset in the GEO database is: GSE116508.
